# Supplementary material for: Canonical MicroRNA Activity Facilitates but May Be Dispensable for Transcription Factor-Mediated Reprogramming
Source: Stem Cell Reports. 2015 Dec 8;5(6):1119–27. doi: 10.1016/j.stemcr.2015.11.002 (PMC4682342; doi:10.1016/j.stemcr.2015.11.002)
Supplement: Document S1. Figures S1–S3 and Tables S1 and S2 [file mmc1.pdf]

Stem Cell Reports, Volume *5*

Supplemental Information

Canonical MicroRNA Activity Facilitates but May Be Dispensable for Transcription Factor-Mediated Reprogramming

Zhong Liu, Maria Skamagki, Kitai Kim, and Rui Zhao

EXTENDED FIGURE LEGEND

Figure S1. Schematic of the canonical miRNA, endo-shRNA, mirtron, and endo-siRNA biogenesis pathways, related to Figure 1-5.

Figure S2. PCR genotyping of *Dgcr8*$^{\Delta/\Delta}$ NSC-derived iPSC clones isolated from three independent reprogramming experiments, related to Figure 2. Note that *Dgcr8*^{flox} alleles were completely disrupted in all analyzed iPSC clones. Arrow, *Dgcr8*^{flox}; arrow head, *Dgcr8*$^{\Delta}$.

Figure S3. Characterization of *Dgcr8*$^{\Delta/\Delta}$ iPSCs, related to Figure 3.

(A) The NSC-derived *Dgcr8*$^{\Delta/\Delta}$ iPSCs expressed pluripotency-associated markers (a) alkaline phosphatase, (b) SSEA-1 (red), and (c) NANOG; and (c') DAPI. Scale bars, 100 μm.

(B-C) Additional karyotypes of 40, XY *Dgcr8*$^{\Delta/\Delta}$ iPSCs.

(D) Characterization of transgene-free *Dgcr8*$^{\Delta/\Delta}$ iPSCs. (a) Bright field and (a') YFP. (b-d') The transgene-free *Dgcr8*$^{\Delta/\Delta}$ iPSCs expressed pluripotency-associated markers (b) alkaline phosphatase, (c) SSEA-1, and (d) NANOG; and (d') DAPI. Scale bars, 100 μm (white) and 50 μm (green).

Figure S1

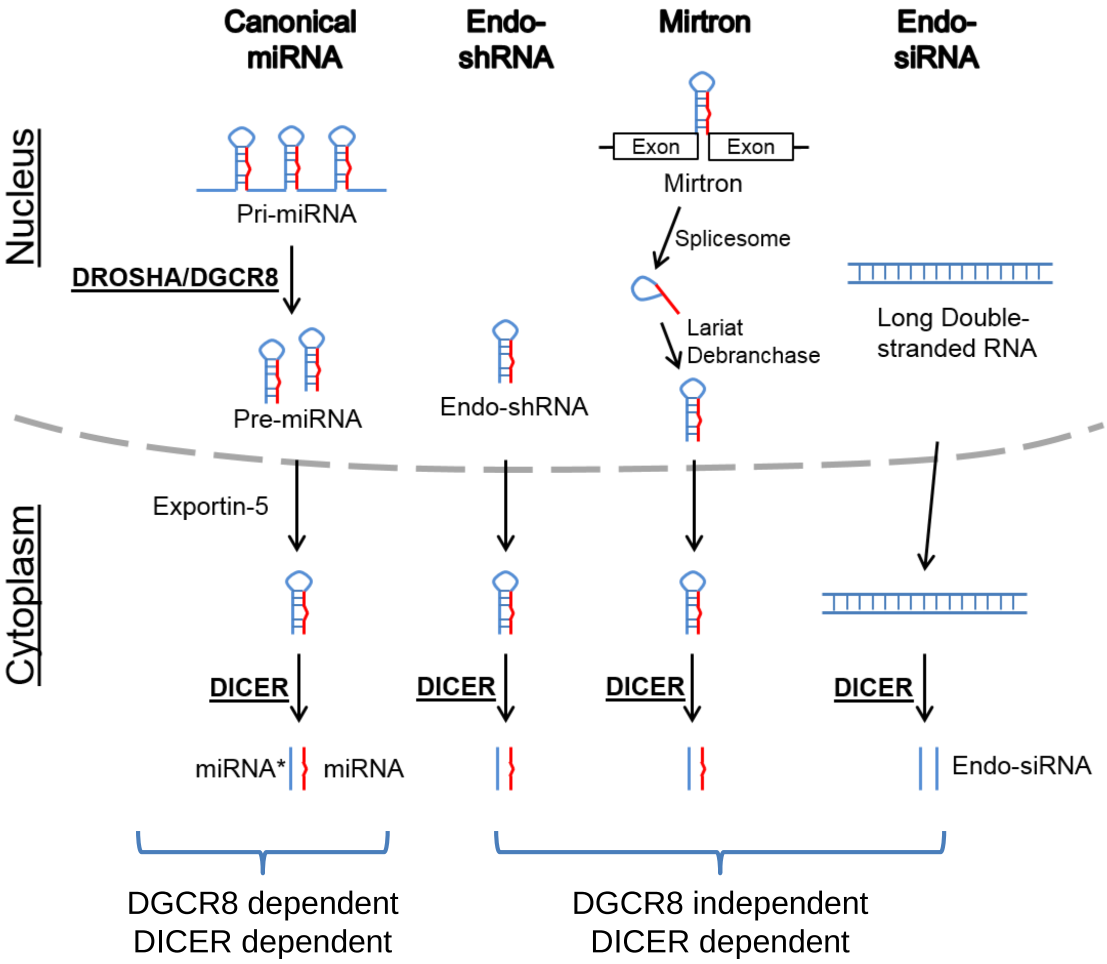

Canonical miRNA
Endo-shRNA
Mirtron
Endo-siRNA
Nucleus
Cytoplasm
Pri-miRNA
DROSHA/DGCR8
Pre-miRNA
Exportin-5
DICER
miRNA*
miRNA
Endo-shRNA
Exon
Exon
Mirtron
Splicesome
Lariat
Debranchase
Long Double-stranded RNA
Endo-siRNA
DGCR8 dependent
DICER dependent
DGCR8 independent
DICER dependent

Figure S2.

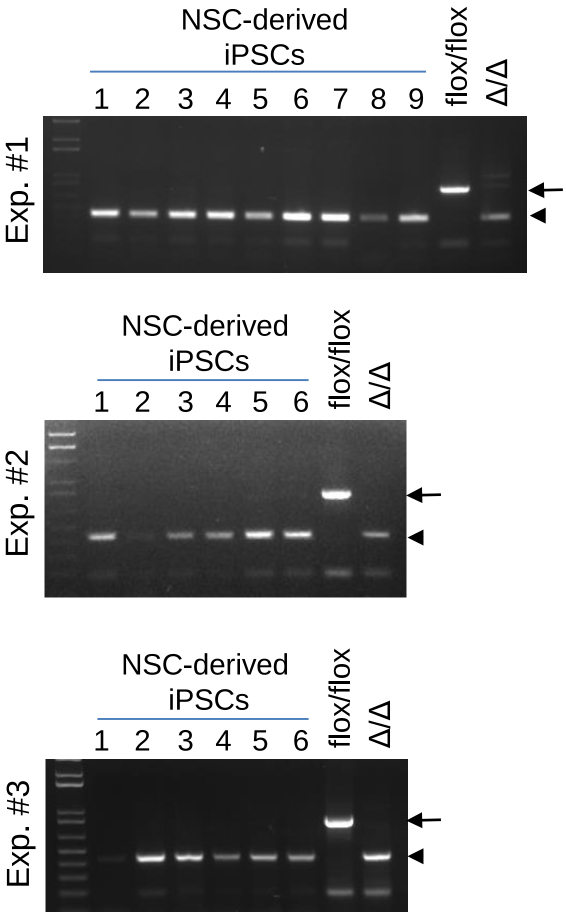
NSC-derived iPSCs
1 2 3 4 5 6 7 8 9
flox/flox
Δ/Δ
Exp. #1
NSC-derived iPSCs
1 2 3 4 5 6
flox/flox
Δ/Δ
Exp. #2
NSC-derived iPSCs
1 2 3 4 5 6
flox/flox
Δ/Δ
Exp. #3

Figure S3.

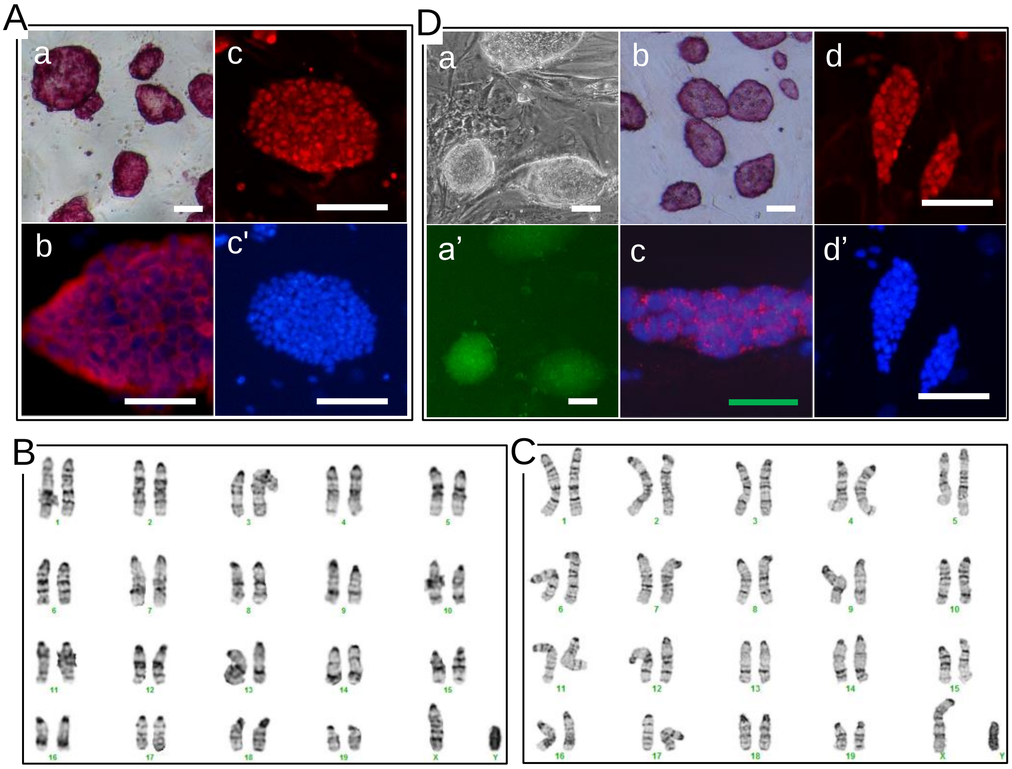
A
a
c
b
c'
D
a
b
d
a'
c
d'
B
1
2
3
4
5
6
7
8
9
10
11
12
13
14
15
16
17
18
19
X
Y
C
1
2
3
4
5
6
7
8
9
10
11
12
13
14
15
16
17
18
19
X
Y

Table S1. Frequency of incomplete *Dgcr8* deletion in *Dgcr8*$^{\Delta/\Delta}$ fibroblast reprogramming, related to Figure 1.

	**# of input fibroblasts**	**# of iPS colonies**	**# of *Dgcr8*$^{\Delta/\Delta}$ iPS colonies**	**# of incomplete deleted colonies**	**Reprog. efficiency (%)***
Exp. 1	50,000	2	2	0	0.004
Exp. 2	50,000	2	2	0	0.004
Exp. 3	50,000	11	9	2	0.018
Exp. 4	50,000	6	5	1	0.010
Exp. 5	50,000	1	1	0	0.002
Total		22	19	3	
% total			86.4	13.6	

*Reprogramming efficiency is determined by the ratio of *Dgcr8*$^{\Delta/\Delta}$ iPS colonies to input fibroblasts.

Table S2. Primer sequences, related to Figure 3 and 4.

**Gene**	**Primers**	**Sequence**
Oct4	Oct4f<br>Oct4r	5′ – AGC TGC TGA AGC AGA AGA GGA TCA – 3′<br>5′ – TCT CAT TGT TGT CGG CTT CCT CCA – 3′
Sox2	Sox2f<br>Sox2r	5′ – CAC ATG AAG GAG CAC CCG GAT TAT – 3′<br>5′ – TCC GGG AAG CGT GTA CTT ATC CTT – 3′
Nanog	Nanogf<br>Nanogr	5′ – AAC CAA AGG ATG AAG TGC AAG CGG – 3′<br>5′ – TCC AAG TTG GGT TGG TCC AAG TCT – 3′
Sox1	Sox1f<br>Sox1r	5′ – CCA AGA TGC ACA ACT CGG AGA TCA – 3′<br>5′ – ACT TGT CCT TCT TGA GCA GCG TCT – 3′
Fgf5	Fgf5f<br>Fgf5r	5′ – ATA GCA GTT TCC AGT GGA GCC CTT – 3′<br>5′ – ACT TAA CAC ACT GGC TTC GTG GGA – 3′
Krt18	Krt18f<br>Krt18r	5′ – ATC TGG AGT CAG AGC TGG CAC AAA – 3′<br>5′ – TCC AGG GCA TCG TTG AGA CTG AAA – 3′
Brachyury	Brach-f<br>Brach-r	5′ – AGC TCT CCA ACC TAT GCG GAC AAT – 3′<br>5′ – TGG TAC CAT TGC TCA CAG ACC AGA – 3′
Afp	Afp-f<br>Afp-r	5′ – AGT TTC CAG AAC CTG CCG AGA GTT – 3′<br>5′ – TGG AAG CAC TCC TCC TTG TTG TCA – 3′
Hnf4a	Hnf4a-f<br>Hnf4a-r	5′ – TTC GGC ATG GCC AAG ATT GAC AAC – 3′<br>5′ – TTG GTG CCC ATG TGT TCT TGC ATC – 3′
Eomes	Eomes-f<br>Eomes-r	5′ – AAG GCT TCC GGG ACA ACT ACG ATT – 3′<br>5′ – TGA GGC AAA GTG TTG ACA AAG GGC – 3′
Dgcr8	Dgcr8f<br>Dgcr8r	5′ – GTG GAT GAA GAG GCC TTG AA – 3′<br>5′ – TCT TGG GAG CAC AGA GAC CT – 3′
Oct4-tg	hOct4-tg-F<br>F2A-R	5′ – GCT CTC CCA TGC ATT CAA AC – 3′<br>5′ – TTG GAC CTG GAT TTG ACT CTA C – 3′
Sox2-tg	hSox2F<br>hSox2R	5′ – CAC ATG AAG GAG CAC CCG GAT TAT – 3′<br>5′ – GTT CAT GTG CGC GTA ACT GTC CAT – 3′
b-actin	bActin-f<br>bActin-r	5′ – TTG CTG ACA GGA TGC AGA AGG AGA – 3′<br>5′ – ACT CCT GCT TGC TGA TCC ACA TCT – 3′
Lentiviral transgene	hOct4-tg-F<br>hKlf4-tg-R	5′ – TGT ACT CCT CGG TCC CTT T – 3′<br>5′ – TGC TTG ACG CAG TGT CTT – 3′
Genomic control	gDgcr8-P1f<br>gDgcr8-P1r	5′ – TTT CCA ACC CAA GTC AGC AGA T – 3′<br>5′ – AGT GCA TGT GCC ATG CTG CCA – 3′
