# Supplementary material for: Canonical MicroRNA Activity Facilitates but May Be Dispensable for Transcription Factor-Mediated Reprogramming
Source: Stem Cell Reports. 2015 Dec 8;5(6):1119–27. doi: 10.1016/j.stemcr.2015.11.002 (PMC4682342; doi:10.1016/j.stemcr.2015.11.002)
Supplement: Document S2. Article plus Supplemental Information [file mmc2.pdf]

Stem Cell Reports
Article

OPEN ACCESS

Canonical MicroRNA Activity Facilitates but May Be Dispensable for Transcription Factor-Mediated Reprogramming

Zhong Liu,[1] Maria Skamagki,[2] Kitai Kim,[2,*] and Rui Zhao[1,*]
[1]Department of Biochemistry and Molecular Genetics, Stem Cell Institute, University of Alabama at Birmingham, Birmingham, AL 35294, USA
[2]Cancer Biology and Genetics Program, Center for Cell Engineering, Center for Stem Cell Biology, Sloan-Kettering Institute, Cell and Developmental Biology Program, Weill Medical College of Cornell University, New York, NY 10065, USA
*Correspondence: kimk@mskcc.org (K.K.), ruizhao@uab.edu (R.Z.)
http://dx.doi.org/10.1016/j.stemcr.2015.11.002
This is an open access article under the CC BY license (http://creativecommons.org/licenses/by/4.0/).

SUMMARY

MicroRNAs (miRNAs) are important regulators of reprogramming of somatic cells into induced pluripotent stem cells (iPSCs); however, it is unclear whether miRNAs are required for reprogramming and whether miRNA activity as a whole facilitates reprogramming. Here we report on successful reprogramming of mouse fibroblasts and neural stem cells (NSCs) lacking *Dgcr8*, a factor required for the biogenesis of canonical miRNAs, by Yamanaka factors, albeit at decreased efficiencies. Though iPSCs derived from *Dgcr8*-deficient mouse fibroblasts or NSCs were able to self-renew and expressed pluripotency-associated markers, they exhibited poor differentiation potential into mature somatic tissues, similar to *Dgcr8*$^{-/-}$ embryonic stem cells. The differentiation defects could be rescued with expression of *DGCR8* cDNA. Our data demonstrate that while miRNA activity as a whole facilitates reprogramming, canonical miRNA may be dispensable in the derivation of iPSCs.

INTRODUCTION

MicroRNAs (miRNAs) are short, endogenous, non-coding RNAs that repress gene expression post-transcriptionally by destabilizing and/or repressing translation of target mRNAs. In the canonical biogenesis pathway, primary microRNA transcripts (pri-miRNAs) are processed in the nucleus by the microprocessor complex, which consists of the RNase III enzyme DROSHA and the double-stranded RNA-binding protein DGCR8, to generate ~70-nt precursor miRNAs (pre-miRNAs). The pre-miRNAs are then exported to the cytoplasm by EXPORTIN-5 and further processed by another RNase III enzyme, DICER, to generate ~22-nt mature miRNAs (Figure S1) (Kim et al., 2009). More than 400 miRNAs have been identified in the human (Landgraf et al., 2007), and up to 60% of all human genes may be regulated by miRNAs (Friedman et al., 2009).

Given the potentially vast regulatory influence of miRNAs on gene expression and the critical roles of these molecules in embryo development (Bartel, 2009; Sun and Lai, 2013), it is not surprising that miRNAs have emerged as important regulators in reprogramming somatic cells into induced pluripotent stem cells (iPSCs). Together with the Yamanaka factors (OCT4, SOX2, KLF4, and c-MYC) (Takahashi and Yamanaka, 2006), co-expression of the miRNA cluster 302/367 or 106a/363; members of the miR-302, miR-294, or miR-181 family; or miR-93 and miR-106b greatly enhance iPSC derivation efficiency (Judson et al., 2013; Li et al., 2011; Liao et al., 2011; Lin et al., 2011; Subramanyam et al., 2011). Furthermore, expression of the miR-302/367 cluster or miR-200c, miR-302, and miR-369 without the Yamanaka factors is sufficient to reprogram human and mouse fibroblasts (Anokye-Danso et al., 2011; Miyoshi et al., 2011). How these miRNAs promote reprogramming is only partially understood. Several mechanisms have been proposed, such as acceleration of mesenchymal to epithelial transition and antagonism of the activities of let-7 family miRNAs, MBD2, NR2F2, and/or other reprogramming suppressors (Hu et al., 2013; Judson et al., 2013; Lee et al., 2013; Liao et al., 2011; Melton et al., 2010).

In addition to the miRNAs that promote reprogramming, several miRNAs that inhibit reprogramming, such as the let-7 family members, have been reported (Melton et al., 2010; Unternaehrer et al., 2014). Therefore, it remains unclear whether miRNA activity as a whole facilitates reprogramming and whether miRNAs are required to convert somatic cells into iPSCs. Previous attempts to reprogram *Dicer* null mouse embryonic fibroblasts (MEFs) were unsuccessful (Kim et al., 2012); however, this observation cannot rule out a requirement of miRNAs in reprogramming because DICER is also critical for the biogenesis of several other small RNAs, such as endogenous small hairpin RNAs (shRNAs), mirtrons, and endogenous small interfering RNAs (siRNAs) (Figure S1) (Babiarz et al., 2008). In this study, we addressed the question of whether miRNAs are required for generating iPSC by reprogramming mouse cells that lack *Dgcr8*, a factor required specifically for the biogenesis of canonical miRNAs (Figure S1), including all miRNAs implicated in reprogramming (Babiarz et al., 2008; Judson et al., 2013; Wang et al., 2007). We report that *Dgcr8*-deficient fibroblasts and NSCs can be reprogrammed by the Yamanaka factors, albeit at decreased

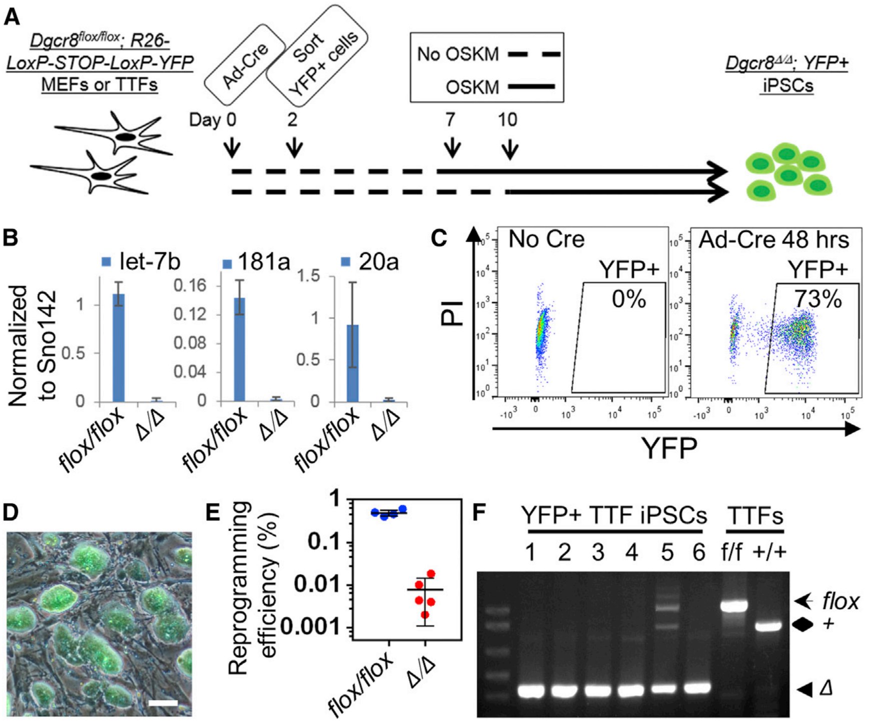

Figure 1. Reprogramming of *Dgcr8*$^{\Delta/\Delta}$ MEFs and TTFs

(A) Schematic of the reprogramming strategy. R26-loxP-STOP-loxP-YFP, ROSA26-driven loxP-flanked STOP sequence followed by an YFP reporter; Ad-Cre, Cre-expressing adenovirus; OSKM, reprogramming factors OCT4, SOX2, KLF4, and c-MYC.

(B) QPCR analyses of mature miRNAs in *Dgcr8*$^{flox/flox}$ and *Dgcr8*$^{\Delta/\Delta}$ TTFs 7 or 10 days after Cre expression. Shown are tested miRNAs reliably expressed in *Dgcr8*$^{flox/flox}$ TTFs. Expression of mature miRNA was normalized to small nucleolar RNA 142. n = 3 independent biological repeats. Error bar, SD.

(C) Representative flow cytometry analysis of the *Dgcr8*$^{\Delta/\Delta}$;*LoxP-STOP-LoxP-YFP* fibroblasts 48 hr after mock (left) or Cre adenovirus (right) transduction. PI, propidium iodide.

(D) Merged bright field and YFP image of fibroblast-derived *Dgcr8*$^{\Delta/\Delta}$ iPSCs. Scale bars, 100 μm.

(E) Reprogramming efficiency of *Dgcr8*$^{flox/flox}$ and *Dgcr8*$^{\Delta/\Delta}$ fibroblasts. n = 4 or 5 independent biological repeats. Error bar, SD. See also Table S1.

(F) PCR genotyping of wild-type, *Dgcr8*$^{flox/flox}$ TTFs, and *Dgcr8*$^{\Delta/\Delta}$ TTF-derived iPSC clones derived from a representative reprogramming experiment. Although most iPSC clones have *Dgcr8* disrupted completely, approximately 15% of YFP+ clones, such as iPSC-5, retain one functional *Dgcr8* allele. Diamond, *Dgcr8*$^{+}$; arrow, *Dgcr8*^{flox}; arrowhead, *Dgcr8*$^{\Delta}$.

efficiencies. These results demonstrate that while canonical miRNAs as a whole facilitate reprogramming, they may be dispensable for the derivation of iPSCs.

RESULTS

Reprogramming of *Dgcr8*$^{\Delta/\Delta}$ MEFs and Tail Tip Fibroblasts

To assess the requirement of miRNAs in iPSC derivation, we first tested whether *Dgcr8*-deficient MEFs and tail tip fibroblasts (TTFs) could be reprogrammed by Yamanaka factors. Because *Dgcr8* null embryos become grossly malformed by embryonic day (E) 6.5 and absorbed by E10 (Wang et al., 2007), isolation of MEFs or TTFs from *Dgcr8* null mice was not possible. Instead, we obtained *Dgcr8*$^{\Delta/\Delta}$ fibroblasts by Cre-mediated disruption of *Dgcr8* in *Dgcr8*$^{flox/flox}$ MEFs or TTFs (Figure 1A) (Suh et al., 2010; Wang et al., 2007). To monitor Cre activity and enable purification of *Dgcr8*$^{\Delta/\Delta}$ fibroblasts, we isolated MEFs or TTFs from *Dgcr8*$^{flox/flox}$ mice carrying a ROSA26-LoxP-STOP-LoxP-YFP (R26-LSL-YFP) reporter (Srinivas et al., 2001). A previous report demonstrated that mature miRNAs are eliminated in *Dicer*$^{\Delta/\Delta}$ MEFs by 6 days after transduction of Cre-expressing lentivirus (Kim et al., 2012). To measure the levels of mature miRNAs after *Dgcr8* disruption, we performed qPCR analyses on *Dgcr8*$^{flox/flox}$ and *Dgcr8*$^{\Delta/\Delta}$ TTFs 7 and 10 days after Cre expression. Among the miRNAs examined, we found that let-7b, miR-20a, and miR-181a were reliably expressed in the *Dgcr8*$^{flox/flox}$ TTFs, but expression of all three miRNAs was reduced to negligible levels in the *Dgcr8*$^{\Delta/\Delta}$ TTFs (Figure 1B), which is consistent with the previous report (Kim et al., 2012). To ensure that only *Dgcr8*$^{\Delta/\Delta}$ fibroblasts were used for reprogramming and to exclude those cells that may disrupt *Dgcr8* during reprogramming, we sorted YFP+ cells 48 hr after transduction of the Cre adenovirus (Figures 1A and 1C). The sorted YFP+ cells were then cultured to 7 or 10 days after Cre adenovirus transduction to deplete miRNAs (Figure 1A). The resulting cells were transduced with STEMCCA lentivirus, which expresses all four Yamanaka factors in a single polycistronic transcript (Somers et al., 2010), to generate iPSCs (Figure 1A). Both *Dgcr8*$^{\Delta/\Delta}$ MEFs and TTFs yielded iPSC colonies in 3 weeks (Figure 1D) at reprogramming efficiencies of 0.002%–0.02%, which was significantly lower than the 0.4%–0.6% efficiency of control *Dgcr8*$^{flox/flox}$ fibroblasts (Figure 1E). Genotyping

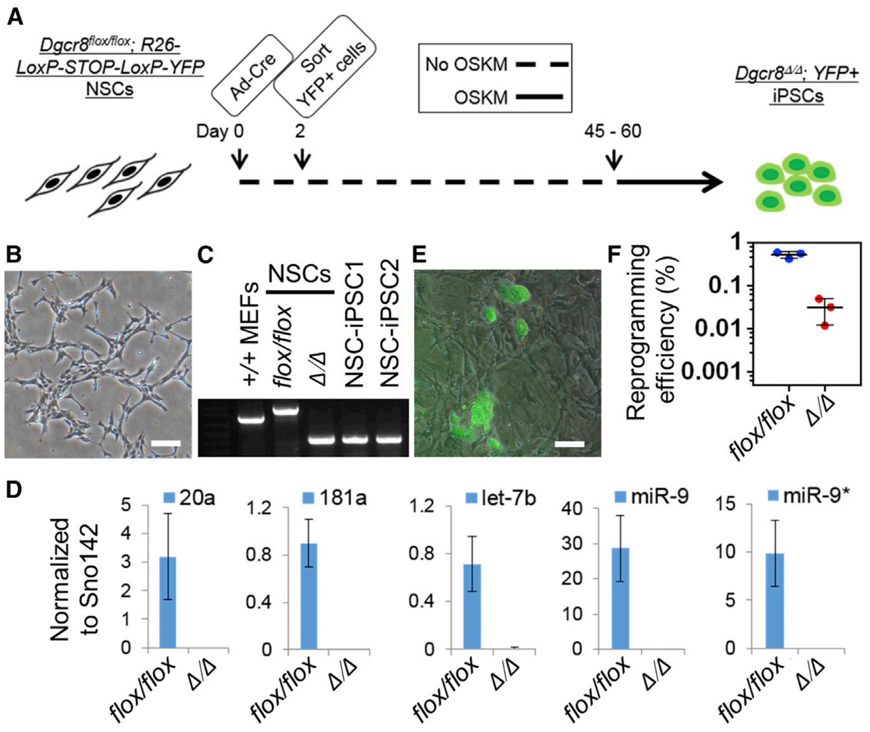

Figure 2. Reprogramming of *Dgcr8*$^{\Delta/\Delta}$ NSCs

(A) Schematic of the reprogramming strategy. R26-loxP-STOP-loxP-YFP, ROSA26-driven loxP-flanked STOP sequence followed by an YFP reporter; Ad-Cre, Cre-expressing adenovirus; OSKM, reprogramming factors OCT4, SOX2, KLF4, and c-MYC.

(B) Bright field image of *Dgcr8*$^{\Delta/\Delta}$ NSCs continuously cultured for 60 days. Scale bars, 100 μm.

(C) PCR genotyping of wild-type MEFs, *Dgcr8*$^{flox/flox}$ NSCs, *Dgcr8*$^{\Delta/\Delta}$ NSCs, and representative *Dgcr8*$^{\Delta/\Delta}$ NSC-derived iPSC clones. See also Figure S2.

(D) QPCR analyses of mature miRNAs in *Dgcr8*$^{flox/flox}$ and *Dgcr8*$^{\Delta/\Delta}$ NSCs. Expression of mature miRNA was normalized to small nucleolar RNA 142. n = 3 independent biological repeats. Error bar, SD.

(E) Merged bright field and YFP image of NSC-derived *Dgcr8*$^{\Delta/\Delta}$ iPSCs. Scale bars, 100 μm.

(F) Reprogramming efficiency of *Dgcr8*$^{flox/flox}$ and *Dgcr8*$^{\Delta/\Delta}$ NSCs. n = 3 independent biological repeats. Error bar, SD.

confirmed that the majority of the resulting iPSCs had both *Dgcr8* alleles disrupted; however, approximately 15% of YFP+ iPSCs retained one functional allele of *Dgcr8*, suggesting that the R26-LSL-YFP reporter is imperfect in monitoring disruption of endogenous genes and that those fibroblasts expressing a single *Dgcr8* allele would have a reprogramming advantage (Figure 1F; Table S1).

Reprogramming of *Dgcr8*$^{\Delta/\Delta}$ Mouse Neural Stem Cells

Though the miRNAs in *Dgcr8*$^{\Delta/\Delta}$ fibroblasts were under the qPCR detection limit (Figure 1B), we could not exclude the possibility that a residual amount of miRNAs remains in a small percentage of fibroblasts 7–10 days after Cre transduction and is required for reprogramming. *Dgcr8*$^{\Delta/\Delta}$ fibroblasts quickly deteriorate in culture (data not shown), which precludes long-term passaging to eliminate any residual miRNAs through cell division-mediated dilution and miRNA degradation. In contrast, neural stem cells (NSCs) can be cultured long term in vitro (Andersson et al., 2010; Kawase-Koga et al., 2010), so we used *Dgcr8*$^{flox/flox}$ NSCs to further examine the requirement of miRNAs in reprogramming (Figure 2A). We isolated NSCs from brains of E13.5 *Dgcr8*$^{flox/flox}$; *R26-LSL-YFP* mice and disrupted *Dgcr8* by transduction of Cre adenovirus (Figure 2A). YFP+ NSCs underwent fluorescence-activated cell sorting (FACS) 48 hr after Cre transduction to exclude cells that had not yet activated Cre. We continuously cultured the sorted *Dgcr8*$^{\Delta/\Delta}$ NSCs for 45–60 days (9–12 passages) (Figure 2B) to ensure exhaustion of any residual miRNAs by cell division-mediated dilution and degradation. PCR-based genotyping analysis detected no contamination of cells with incomplete *Dgcr8* disruption in the prolonged culture of *Dgcr8*$^{\Delta/\Delta}$ NSCs (Figure 2C). The qPCR analysis confirmed that *Dgcr8*$^{\Delta/\Delta}$ NSCs did not express mature miRNAs such as miR-20a, miR-181a, let-7b, and miR-9/9*, which are abundantly expressed in *Dgcr8*$^{flox/flox}$ NSCs (Figure 2D). The resulting *Dgcr8*$^{\Delta/\Delta}$ NSCs were then transduced with STEMCCA lentivirus to generate iPSCs. The control *Dgcr8*$^{flox/flox}$ NSCs were reprogrammed at an efficiency of 0.5%, which is comparable to published data (Kim et al., 2008). We detected YFP+ iPSC colonies 4 weeks after STEMCCA transduction of *Dgcr8*$^{\Delta/\Delta}$ NSCs at efficiencies of 0.01%–0.05% (Figures 2E and 2F). Genotyping of the resulting iPSCs confirmed that *Dgcr8* was disrupted in all examined clones (Figure 2C; Figure S2).

Characterization of *Dgcr8*$^{\Delta/\Delta}$ iPSCs

The *Dgcr8*$^{\Delta/\Delta}$ iPSCs derived from fibroblasts or NSCs expressed pluripotency-associated markers such as alkaline phosphatase (AP), SSEA-1, and NANOG (Figures 3A–3C′; Figure S3A). The qPCR analysis confirmed the lack of *Dgcr8* expression in *Dgcr8*$^{\Delta/\Delta}$ iPSCs (Figure 3D). Karyotyping analyses demonstrated that normal *Dgcr8*$^{\Delta/\Delta}$ iPSCs could be isolated (Figure 3E; Figures S3B and S3C). The qPCR analyses revealed that Yamanaka factors delivered

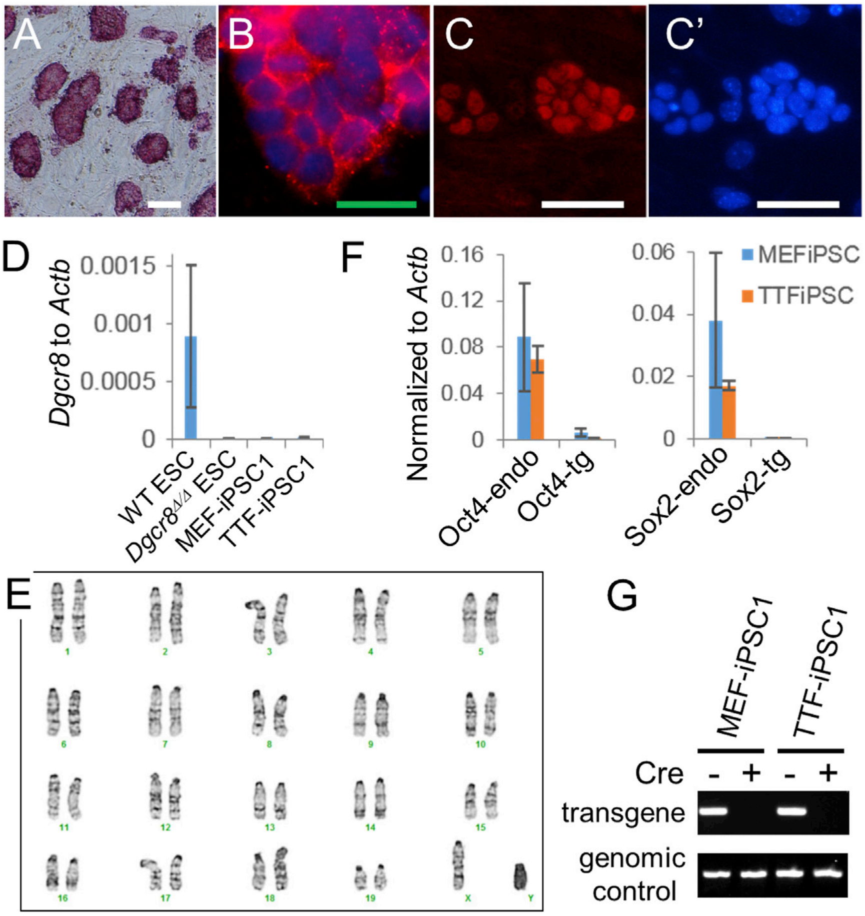

Figure 3. Characterization of $Dgcr8^{\Delta/\Delta}$ iPSCs

(A–C′) $Dgcr8^{\Delta/\Delta}$ iPSCs expressed pluripotency-associated markers. (A) AP, (B) SSEA-1 (red) and DAPI (blue), (C) NANOG, and (C′) DAPI. Scale bars, 100 μm (white) and 50 μm (green). See also Figure S3A for characterization of NSC-derived $Dgcr8^{\Delta/\Delta}$ iPSCs.

(D) QPCR analyses of *Dgcr8* in wild-type ESCs, $Dgcr8^{\Delta/\Delta}$ ESCs, and $Dgcr8^{\Delta/\Delta}$ iPSC clones derived from MEFs or TTFs. Data were normalized to the mRNA levels of β-actin gene *Actb*. n = 3 independent biological repeats. Error bar, SD.

(E) A normal karyotype (40, XY) of $Dgcr8^{\Delta/\Delta}$ iPSCs. See also Figures S3B and S3C.

(F) QPCR analyses of *Oct4* (left) and *Sox2* (right) in representative $Dgcr8^{\Delta/\Delta}$ iPSC clones derived from MEFs or TTFs. Endo, endogenous expression; tg, transgene expression. Data were normalized to the mRNA levels of β-actin gene *Actb*. n = 3 independent biological repeats. Error bar, SD.

(G) PCR confirmation of transgene-free $Dgcr8^{\Delta/\Delta}$ iPSC clones. The STEMCCA lentivirus in representative $Dgcr8^{\Delta/\Delta}$ iPSC clones was removed by Cre adenovirus transduction. See also Figure S3D for characterization of the transgene-free $Dgcr8^{\Delta/\Delta}$ iPSCs.

by the STEMCCA lentivirus were largely silenced in $Dgcr8^{\Delta/\Delta}$ iPSCs (Figure 3F). Furthermore, transgene-free $Dgcr8^{\Delta/\Delta}$ iPSCs could be isolated and stably maintained after removal of the STEMCCA lentivirus by Cre adenovirus transduction (Figure 3G; Figure S3D) (Somers et al., 2010).

Next, we evaluated the differentiation capacity of $Dgcr8^{\Delta/\Delta}$ iPSCs in embryoid bodies (EBs). EBs of $Dgcr8^{\Delta/\Delta}$ iPSCs failed to form cystic cavities over an 11-day period, suggesting a lack of differentiation (Figures 4A and 4B). The qPCR analyses revealed that pluripotency-associated markers *Oct4* and *Nanog* were maintained but lineage-specific markers, such as *Fgf5* and *Krt18* (ectodermal), *Brachyury* (mesodermal), *Afp* and *Hnf4a* (endodermal), and *Eomes* (extraembryonic), were weakly expressed or absent in EBs of $Dgcr8^{\Delta/\Delta}$ iPSCs. The only gene modestly upregulated in EBs of $Dgcr8^{\Delta/\Delta}$ iPSCs was *Sox1* (Figure 4C), which is expressed by neural progenitor cells (Ying et al., 2003). To test whether $Dgcr8^{\Delta/\Delta}$ iPSCs could differentiate into more mature neuronal cells, we extended the differentiation protocol under pro-neuronal conditions. Unlike wild-type embryonic stem cells (ESCs), mature Tuj1+ neurons were not differentiated from $Dgcr8^{\Delta/\Delta}$ iPSCs (Figures 4D and 4E). These data are consistent with the previous finding that $Dgcr8^{-/-}$ ESCs poorly produce mature somatic cells (Wang et al., 2007).

Next, we restored DGCR8 expression to levels similar to wild-type ESCs using a human *DGCR8* cDNA (Figure 5A). The DGCR8-rescued iPSCs exhibited an accelerated cell cycle with a shortened G1 phase compared to $Dgcr8^{\Delta/\Delta}$ iPSCs (Figure 5B), which underwent slower proliferation, similar to $Dgcr8^{-/-}$ ESCs (Wang et al., 2008). To test whether DGCR8 rescue restored the differentiation potential of the $Dgcr8^{\Delta/\Delta}$ iPSCs, we performed a colony-forming assay to examine the number of differentiation-resistant cells within the $Dgcr8^{\Delta/\Delta}$ and DGCR8-rescued iPSCs. Mutant and rescued iPSCs were first induced to differentiation by retinoic acid and then plated back to conditions supporting self-renewal of iPSCs to form colonies. We found that significantly more colonies were formed by $Dgcr8^{\Delta/\Delta}$ iPSCs than by wild-type control ESCs and DGCR8-rescued iPSCs (Figure 5C). We further evaluated the differentiation potential of rescued iPSCs in a teratoma assay. When injected into immunodeficient mice, the $Dgcr8^{\Delta/\Delta}$ iPSCs formed tumors containing predominantly undifferentiated cells

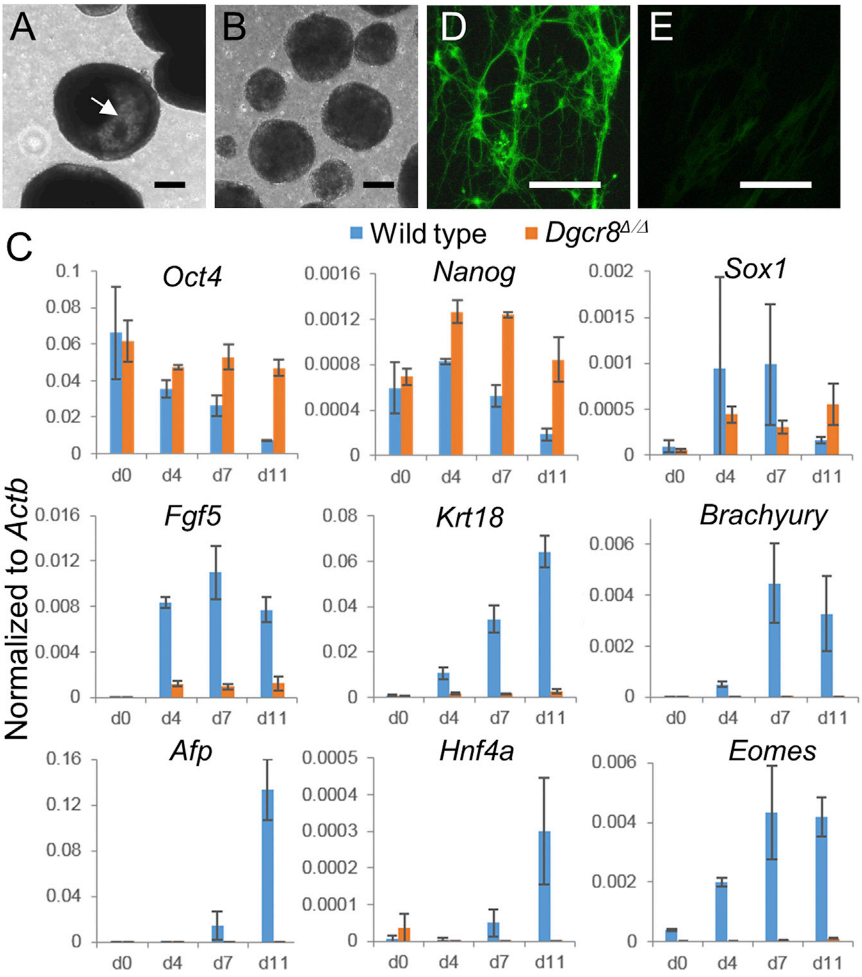

Figure 4. *Dgcr8*$^{\Delta/\Delta}$ iPSCs Are Deficient in Differentiation

(A and B) EBs formed by (A) wild-type ESCs and (B) *Dgcr8*$^{\Delta/\Delta}$ iPSCs. The arrow points to a cystic cavity of an EB. Scale bar, 200 μm.
(C) QPCR analyses of EBs formed by wild-type ESCs and *Dgcr8*$^{\Delta/\Delta}$ iPSCs. The analyzed markers include *Oct4* and *Nanog* (pluripotency associated); *Sox1*, *Fgf5*, and *Krt18* (ectodermal); *Brachyury* (mesodermal); *Afp* and *Hnf4a* (endodermal); and *Eomes* (trophectodermal). Samples were collected at indicated days of differentiation. Data were normalized to the mRNA levels of β-actin gene *Actb*. n = 3 independent biological repeats. Error bar, SD.
(D and E) Immunostaining of Tuj1, a marker specifically expressed by neurons, in EBs of (D) wild-type and (E) *Dgcr8*$^{\Delta/\Delta}$ iPSCs. Scale bar, 100 μm.

(Figure 5D). In contrast, the teratoma formed by DGCR8-rescued iPSCs consisted of tissues from all three embryonic layers (Figures 5E–5E″).

Together, our data support that somatic cells lacking *Dgcr8* and deficient in the biogenesis of canonical miRNAs can be reprogrammed into iPSCs by the Yamanaka factors alone, albeit at decreased reprogramming efficiencies; therefore, canonical miRNA activity facilitates but may be dispensable for iPSC derivation. Consistent with previous reports (Kanellopoulou et al., 2005; Wang et al., 2007), however, miRNAs do appear to be important for subsequent iPSC-derived tissue differentiation.

DISCUSSION

miRNAs may confer robustness to biological systems by integrating into transcriptional regulatory circuitry to reinforce genetic programs and buffer stochastic perturbations (Ebert and Sharp, 2012; Hornstein and Shomron, 2006). Mutant mice with deletions of individual miRNA clusters often exhibit only relatively subtle phenotypic defects (Park et al., 2012). More severe phenotypes are usually observed in mutants with compound deletions of functionally redundant miRNA clusters, suggesting that the subtle defects of individual mutations are at least partially due to functional compensation (Park et al., 2012). The *Dgcr8* and *Dicer* mutants, which have complete miRNA loss, exhibit the most extreme phenotypic defects. The mutant ESCs can self-renew and express stem cell markers but are functionally defective in spontaneous differentiation (Kanellopoulou et al., 2005; Wang et al., 2007). These results suggest that the regulatory circuitry of pluripotent cells can be sustained solely by transcription factors, while miRNAs are required to initiate and/or sustain the differentiation. Our data support this notion. Because reprogramming is generally considered to be a de-differentiation process, our data suggest that miRNA activity may not be essential for de-differentiation but is essential for normal tissue differentiation.

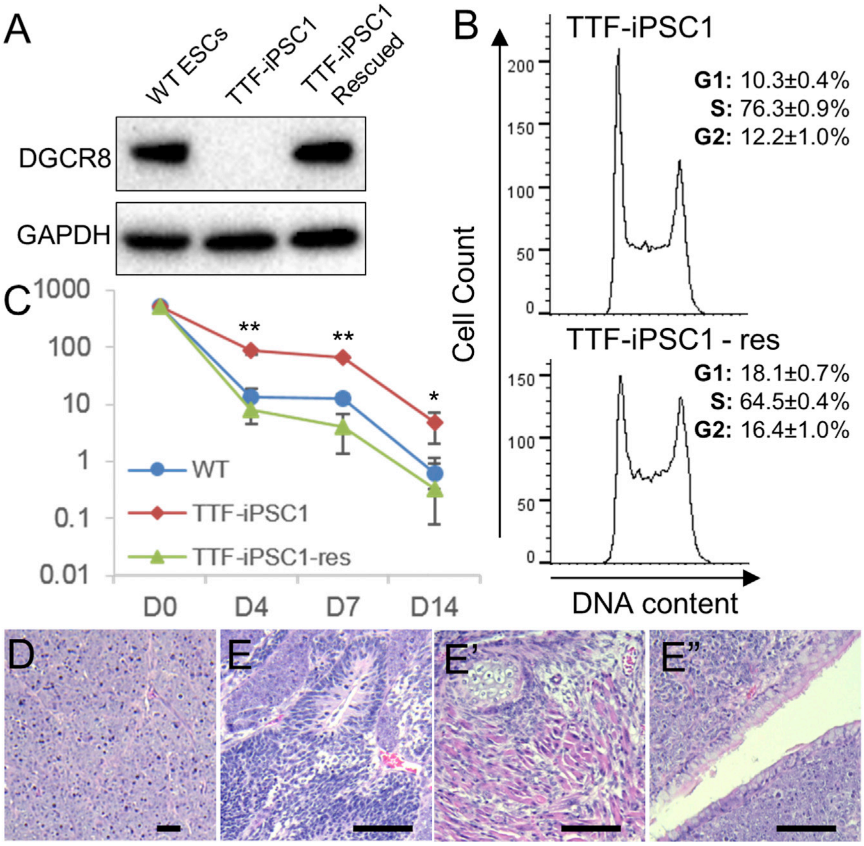

Figure 5. Rescue of DGCR8 Restored Differentiation Potential of *Dgcr8*$^{\Delta/\Delta}$ iPSCs

(A) Immunoblot of DGCR8 (top) and GAPDH (bottom) in wild-type ESC, *Dgcr8*$^{\Delta/\Delta}$ TTF-iPSC, and DGCR8-rescued *Dgcr8*$^{\Delta/\Delta}$ TTF-iPSC extracts.

(B) Cell-cycle analyses of *Dgcr8*$^{\Delta/\Delta}$ iPSCs and rescued *Dgcr8*$^{\Delta/\Delta}$ iPSCs. n = 3 independent biological repeats.

(C) Colony-forming assay of wild-type, *Dgcr8*$^{\Delta/\Delta}$, and DGCR8-rescued *Dgcr8*$^{\Delta/\Delta}$ iPSCs. Cells were first induced to differentiate by treatment with retinoic acid for the indicated days and then returned to conditions permissive to self-renewal for 7 days. Colonies positive for AP were scored. n = 3 independent biological repeats. Error bar, SD. *$p < 0.05$; **$p < 0.01$; Student's t test between *Dgcr8*$^{\Delta/\Delta}$ and rescued iPSCs.

(D–E″) Teratoma analyses. Shown are teratomas generated by (D) *Dgcr8*$^{\Delta/\Delta}$ iPSCs, which contain virtually no differentiated somatic tissues and (E–E″) the DGCR8-rescued *Dgcr8*$^{\Delta/\Delta}$ iPSCs, which contain tissues from all three embryonic germ layers: (E) neural epithelium, (E′) cartilage and muscle, and (E″) respiratory epithelium. Scale bar, 100 μm.

The mechanisms involved in reprogramming somatic cells to iPSCs by the Yamanaka factors remain poorly understood. Because of the low efficiency and slow kinetics of most reprogramming systems, molecular events that direct somatic cells to pluripotency have been difficult to define. Recent work has demonstrated that miRNAs such as miR-294, miR-302, and miR-181 family members facilitate (Judson et al., 2013; Li et al., 2011; Liao et al., 2011; Lin et al., 2011; Melton et al., 2010; Subramanyam et al., 2011), but let-7 family members inhibit, reprogramming (Melton et al., 2010; Unternaehrer et al., 2014). Therefore, it remains unclear whether miRNA activity as a whole promotes reprogramming and whether miRNAs, in particular those miRNAs shown to promote reprogramming, are necessary for the derivation of iPSCs. Here, we present data demonstrating that while miRNA activity as a whole facilitates reprogramming, the derivation of iPSC may be achieved without canonic miRNAs. Because *Dgcr8*$^{\Delta/\Delta}$ fibroblasts do not survive extended culture times, they must be transduced with STEMCCA virus for reprogramming 7 or 10 days after Cre expression. Our qPCR analysis detected negligible levels of miRNAs in these cells (Figure 1B), consistent with a previous report that mature miRNAs are effectively eliminated in *Dicer*$^{\Delta/\Delta}$ MEFs 6 days after transduction of Cre-expressing lentivirus (Kim et al., 2012). Nevertheless, to exclude the possibility that residual miRNAs may be present and essential for reprogramming, we reprogrammed *Dgcr8*$^{\Delta/\Delta}$ NSCs, which can be propagated for longer terms to ensure exhaustion of residual miRNAs before transduction of reprogramming factors (Figure 2A). The prolonged culture of *Dgcr8*$^{\Delta/\Delta}$ NSCs exhausts residual miRNAs by two mechanisms. First, the *Dgcr8*$^{\Delta/\Delta}$ NSCs are proliferative; therefore, residual miRNAs are diluted out with each cell division. We split *Dgcr8*$^{\Delta/\Delta}$ NSCs at a 1:5 ratio for each passage, resulting in the expansion of any single cell to 1.9×10^6–2.4×10^8 (5^9–5^{12}) progeny cells and making it highly unlikely that any residual miRNAs could persist at a biological meaningful concentration by the end of 9–12 passages. Second, the sorted *Dgcr8*$^{\Delta/\Delta}$ NSCs were reprogrammed after a continuous culture for 45–60 days, which is a sufficient duration to achieve complete degradation of residual miRNAs. Therefore, our data conclusively demonstrate that reprogramming of NSCs may be achieved solely by transcriptional factors without any miRNA activities.

Kim et al. (2012) reported that iPSCs could not be isolated from MEFs 6 days after disruption of *Dicer*, which is inconsistent with our data on reprogramming *Dgcr8*$^{\Delta/\Delta}$ fibroblasts (Figure 1). DICER is required for the biogenesis of

not only canonical miRNAs but also other small RNA species, such as endogenous siRNAs, shRNAs, mirtrons, and short interspersed nuclear element-derived RNAs (Figure S1) (Babiarz et al., 2008). The discrepancy between the data on reprogramming of *Dicer*-deficient cells and those of *Dgcr8*-deficient cells probably reflects the activities of some DICER-dependent but DGCR8-independent small RNAs. Alternatively, the poorer proliferation capacity of *Dicer*$^{\Delta/\Delta}$ fibroblasts may contribute to the failure of iPSC derivation (Kim et al., 2012), which is known to be proliferation dependent (Smith et al., 2010). Recently, Zhang et al. (2013) reported that they were unable to isolate iPSCs from human foreskin fibroblasts that were null for the endogenous miR-302/367 cluster. These data suggested the miR-302/367 cluster is required for human somatic cell reprogramming. Although this result is not consistent with our findings, the discrepancy may be explained by the potential difference in somatic cell reprogramming and/or in the self-renewal of human and mouse pluripotent stem cells (Nichols and Smith, 2009). Alternatively, the discrepancy may be caused by the different miRNA deficiencies of the reprogrammed fibroblasts. In our study, the *Dgcr8*$^{\Delta/\Delta}$ fibroblasts lacked miRNAs both promoting reprogramming, such as the miR-290s and miR-302s, and inhibiting reprogramming, such as the let-7s; however, the fibroblasts used by Zhang et al. (2013) were only deficient in the reprogramming-promoting miR-302/367 cluster. The fine balance between pluripotency-promoting and differentiation-inducing miRNAs has been demonstrated to play critical roles in the maintenance of the ground state of pluripotency (Kumar et al., 2014), which could be similarly required in reprogramming. Nonetheless, this is an interesting observation that deserves further investigation.

EXPERIMENTAL PROCEDURES

Mice and the Derivation of ESCs, MEFs, TTFs, and NSCs

All animal experiments were performed in accordance with guidelines from the University of Alabama at Birmingham (UAB) and NIH. *Dgcr8*$^{flox/flox}$; *LSL-YFP* mice were generated by crossing *Dgcr8*$^{flox/flox}$ mice (Wang et al., 2007) and *R26-LSL-YFP* mice (Srinivas et al., 2001). ESCs were derived from E3.5 blastocysts as described (Kim et al., 2010; Liu et al., 2011). MEFs were isolated from E12.5 embryos, and TTFs were derived from 1-week-old mice. NSCs were isolated from brains of E13.5 embryos following a previously published protocol (Currle et al., 2007).

Cell Culture

Mouse ESCs and iPSCs were maintained in mouse ESC maintenance medium (DMEM, 15% fetal bovine serum [FBS; Gemini Bio], 0.1 mM non-essential amino acid [Life Technologies], β-mercaptoethanol [Sigma-Aldrich], and 1,000 U/ml embryonic stem cell growth medium [ESGRO, Millipore]) on gelatin-coated plates as described previously (Kim et al., 2010). For EB differentiation, trypsinized wild-type or mutant iPSCs were suspended in Costar ultra-low-attachment cell culture plates (Corning) at a density of 1×10^5 cells/ml in differentiation medium (ESC maintenance medium without ESGRO). EB samples were collected on the indicated days for total RNA extraction. For neuronal differentiation, EBs (day 4) were plated onto tissue culture plates and cultured in N2 medium (DMEM/F12 and N2 supplement [Gemini Bio]) for up to 25 days. All fibroblasts were cultured in D10 medium (DMEM and 10% FBS). NSCs were cultured in Mouse Neural Stem Cell Expansion medium (EMD Millipore) on tissue culture plates coated with polyornithine (Sigma-Aldrich) and laminin (EMD Millipore).

Lentiviral Production, iPSC Derivation, and Rescue of Dgcr8 Deficiency

Lentivirus expressing STEMCCA (Somers et al., 2010) were prepared as described (Zhao et al., 2014). *Dgcr8*$^{\Delta/\Delta}$ fibroblasts or NSCs were obtained by Cre adenovirus (Vector Biolabs) transduction of *Dgcr8*$^{flox/flox}$; *LSL-YFP* MEFs or TTFs at an MOI of 500 and FACS sorting of YFP+ cells 48 hr after viral transduction. For fibroblast reprogramming, sorted MEFs or TTFs were continuously cultured for seven or ten days before transduction with STEMCCA lentivirus at an MOI of 2. For NSC reprogramming, sorted NSCs were continuously cultured for 45–60 days before transduction with STEMCCA lentivirus at an MOI of 2. The transduced fibroblasts or NSCs were plated directly onto irradiated MEF feeders in mouse ESC maintenance medium (DMEM, 15% FBS, and 1,000 U/ml ESGRO [Millipore]) for up to 4 or 6 weeks, respectively. A human DGCR8 cDNA was subcloned from pFLAG/HA-DGCR8 (Addgene 10921) (Landthaler et al., 2004) into pSIN-EF2-DEST-Pur, a derivative of pSin-EF2-Oct4-Pur (Addgene 16579) (Yu et al., 2007), to generate the lentiviral vector pSIN-EF2-DGCR8-Pur. *Dgcr8*-deficient iPSCs were transduced with lentivirus expressing DGCR8 and selected for puromycin resistance.

Immunostaining, Immunoblotting, and AP Staining

For immunostaining, iPSCs or EBs were fixed in 4% paraformaldehyde, blocked in Protein Block (Dako), and incubated with the appropriate primary antibodies overnight at 4°C and secondary antibodies for 2 hr at room temperature. Images were acquired by a Nikon Ti-S microscope and processed by Photoshop CS6. For immunoblotting, whole cell extracts were prepared in RIPA buffer (50 mM Tris-HCl [pH 8.0], 150 mM NaCl, 1% NP-40, 0.5% sodium deoxycholate, and 0.1% SDS), separated on a 4%–20% SDS-polyacrylamide gel (Bio-Rad), and transferred to polyvinylidene fluoride membrane (Thermo Scientific). Antibodies used were DGCR8 (10996-1-AP, Proteintech), GAPDH (sc-25778, Santa Cruz), SSEA-1 (MC-480, Hybridoma Bank), NANOG (AF2729, R&D Systems), and Tuj1 (801202, BioLegend). For AP staining, cells were fixed in 4% paraformaldehyde and stained using the Leukocyte Alkaline Phosphatase Kit (Sigma-Aldrich).

Genotyping, Karyotyping, and Teratoma Analysis

Genotyping was performed as described (Suh et al., 2010). All cell lines were submitted to Cell Line Genetics for G-band karyotyping. Non-obese diabetic severe combined immunodeficiency

gamma mice 4–8 weeks of age were injected subcutaneously with 1×10^6–5×10^6 iPSCs. Tumors were harvested, fixed with 10% formalin, and processed by the Comparative Pathology Laboratory at UAB or by HistoWiz.

RNA Extraction and qPCR Analyses

Total RNA was isolated with the DirectZol RNA Kit (Zymo Research), and cDNA were synthesized using the Verso cDNA Synthesis Kit (Thermo Scientific). The qPCR was performed using 2x Absolute Blue qPCR Master Mix (Thermo Scientific) on a ViiA 7 real-time PCR system (Life Technologies). Primers are listed in Table S2. The miRNAs were reverse transcribed using the TaqMan MicroRNA Reverse Transcription Kit (Life Technologies). The qPCR was performed using the TaqMan Universal PCR Master Mix and TaqMan MicroRNA Assays for indicated miRNAs (Life Technologies) on the ViiA 7 system.

Colony-Forming Assay

The colony-forming assay was performed as previously described (Wang et al., 2007). In brief, undifferentiated wild-type, *Dgcr8*$^{\Delta/\Delta}$, and rescued *Dgcr8*$^{\Delta/\Delta}$ iPSCs were cultured in differentiation medium supplemented with 2 μM retinoic acid (Sigma-Aldrich) for the indicated days, trypsinized to single cells, replated at a density of 100 cells/cm^2 onto gelatin-coated plates, and cultured in ESC maintenance medium for 7 days before AP staining. Experiments were repeated three times, and only AP-positive colonies were scored.

Cell-Cycle Analysis

Cell-cycle analysis was performed as described (Zhao et al., 2014). In brief, cells at 30%–50% confluency were trypsinized and fixed in cold 70% ethanol at −20°C overnight. Cells were washed twice in PBS, treated with 10 μg/ml DNase-free RNase A at 37°C for 30 min, and resuspended at a density of 5×10^5 cells/ml in PBS with 5 μg/ml propidium iodide. Cells were analyzed on a Becton Dickinson Fortessa flow cytometer, and data were analyzed by the FlowJo VX software.

SUPPLEMENTAL INFORMATION

Supplemental Information includes three figures and two tables and can be found with this article online at http://dx.doi.org/10.1016/j.stemcr.2015.11.002.

ACKNOWLEDGMENTS

We thank Drs. Robert Blelloch (UCSF) for the *Dgcr8*$^{-/-}$ ESCs, Jingyang Feng (Loyola Medical Center) for interpretation of histology results, Tim Townes (UAB) for comments on the manuscript, and the UAB Comprehensive Flow Cytometry Core (P30AR048311 and P30AI027767) for cell sorting. R.Z. is supported by UAB Development Fund, and K.K. is supported by the NIH (5R00HL093212-04 and R01AG043531), TriStem (2013-049), MSK Cancer Center Support Grant/Core Grant (P30CA008748), Louis V. Gerstner Jr. Young Investigators awards, Geoffrey Beene Junior Chair Award, Alfred W. Bressler Scholars Endowment Fund, and Sidney Kimmel Scholars Award.

Received: March 6, 2014
Revised: November 3, 2015
Accepted: November 12, 2015
Published: December 8, 2015

REFERENCES

Andersson, T., Rahman, S., Sansom, S.N., Alsiö, J.M., Kaneda, M., Smith, J., O'Carroll, D., Tarakhovsky, A., and Livesey, F.J. (2010). Reversible block of mouse neural stem cell differentiation in the absence of dicer and microRNAs. PLoS ONE *5*, e13453.

Anokye-Danso, F., Trivedi, C.M., Juhr, D., Gupta, M., Cui, Z., Tian, Y., Zhang, Y., Yang, W., Gruber, P.J., Epstein, J.A., and Morrisey, E.E. (2011). Highly efficient miRNA-mediated reprogramming of mouse and human somatic cells to pluripotency. Cell Stem Cell *8*, 376–388.

Babiarz, J.E., Ruby, J.G., Wang, Y., Bartel, D.P., and Blelloch, R. (2008). Mouse ES cells express endogenous shRNAs, siRNAs, and other Microprocessor-independent, Dicer-dependent small RNAs. Genes Dev. *22*, 2773–2785.

Bartel, D.P. (2009). MicroRNAs: target recognition and regulatory functions. Cell *136*, 215–233.

Currle, D.S., Hu, J.S., Kolski-Andreaco, A., and Monuki, E.S. (2007). Culture of mouse neural stem cell precursors. J. Vis. Exp. *2*, 152.

Ebert, M.S., and Sharp, P.A. (2012). Roles for microRNAs in conferring robustness to biological processes. Cell *149*, 515–524.

Friedman, R.C., Farh, K.K., Burge, C.B., and Bartel, D.P. (2009). Most mammalian mRNAs are conserved targets of microRNAs. Genome Res. *19*, 92–105.

Hornstein, E., and Shomron, N. (2006). Canalization of development by microRNAs. Nat. Genet. Suppl. *38*, S20–S24.

Hu, S., Wilson, K.D., Ghosh, Z., Han, L., Wang, Y., Lan, F., Ransohoff, K.J., Burridge, P., and Wu, J.C. (2013). MicroRNA-302 increases reprogramming efficiency via repression of NR2F2. Stem Cells *31*, 259–268.

Judson, R.L., Greve, T.S., Parchem, R.J., and Blelloch, R. (2013). MicroRNA-based discovery of barriers to dedifferentiation of fibroblasts to pluripotent stem cells. Nat. Struct. Mol. Biol. *20*, 1227–1235.

Kanellopoulou, C., Muljo, S.A., Kung, A.L., Ganesan, S., Drapkin, R., Jenuwein, T., Livingston, D.M., and Rajewsky, K. (2005). Dicer-deficient mouse embryonic stem cells are defective in differentiation and centromeric silencing. Genes Dev. *19*, 489–501.

Kawase-Koga, Y., Low, R., Otaegi, G., Pollock, A., Deng, H., Eisenhaber, F., Maurer-Stroh, S., and Sun, T. (2010). RNAase-III enzyme Dicer maintains signaling pathways for differentiation and survival in mouse cortical neural stem cells. J. Cell Sci. *123*, 586–594.

Kim, B.M., Thier, M.C., Oh, S., Sherwood, R., Kanellopoulou, C., Edenhofer, F., and Choi, M.Y. (2012). MicroRNAs are indispensable for reprogramming mouse embryonic fibroblasts into induced stem cell-like cells. PLoS ONE *7*, e39239.

Kim, J.B., Zaehres, H., Wu, G., Gentile, L., Ko, K., Sebastiano, V., Araúzo-Bravo, M.J., Ruau, D., Han, D.W., Zenke, M., and Schöler, H.R. (2008). Pluripotent stem cells induced from adult neural stem cells by reprogramming with two factors. Nature *454*, 646–650.

Kim, K., Doi, A., Wen, B., Ng, K., Zhao, R., Cahan, P., Kim, J., Aryee, M.J., Ji, H., Ehrlich, L.I., et al. (2010). Epigenetic memory in induced pluripotent stem cells. Nature *467*, 285–290.

Kim, V.N., Han, J., and Siomi, M.C. (2009). Biogenesis of small RNAs in animals. Nat. Rev. Mol. Cell Biol. *10*, 126–139.

Kumar, R.M., Cahan, P., Shalek, A.K., Satija, R., DaleyKeyser, A.J., Li, H., Zhang, J., Pardee, K., Gennert, D., Trombetta, J.J., et al. (2014). Deconstructing transcriptional heterogeneity in pluripotent stem cells. Nature *516*, 56–61.

Landgraf, P., Rusu, M., Sheridan, R., Sewer, A., Iovino, N., Aravin, A., Pfeffer, S., Rice, A., Kamphorst, A.O., Landthaler, M., et al. (2007). A mammalian microRNA expression atlas based on small RNA library sequencing. Cell *129*, 1401–1414.

Landthaler, M., Yalcin, A., and Tuschl, T. (2004). The human DiGeorge syndrome critical region gene 8 and its *D. melanogaster* homolog are required for miRNA biogenesis. Curr. Biol. *14*, 2162–2167.

Lee, M.R., Prasain, N., Chae, H.D., Kim, Y.J., Mantel, C., Yoder, M.C., and Broxmeyer, H.E. (2013). Epigenetic regulation of NANOG by miR-302 cluster-MBD2 completes induced pluripotent stem cell reprogramming. Stem Cells *31*, 666–681.

Li, Z., Yang, C.S., Nakashima, K., and Rana, T.M. (2011). Small RNA-mediated regulation of iPS cell generation. EMBO J. *30*, 823–834.

Liao, B., Bao, X., Liu, L., Feng, S., Zovoilis, A., Liu, W., Xue, Y., Cai, J., Guo, X., Qin, B., et al. (2011). MicroRNA cluster 302–367 enhances somatic cell reprogramming by accelerating a mesenchymal-to-epithelial transition. J. Biol. Chem. *286*, 17359–17364.

Lin, S.L., Chang, D.C., Lin, C.H., Ying, S.Y., Leu, D., and Wu, D.T. (2011). Regulation of somatic cell reprogramming through inducible mir-302 expression. Nucleic Acids Res. *39*, 1054–1065.

Liu, Z., Hu, Z., Pan, X., Li, M., Togun, T.A., Tuck, D., Pelizzola, M., Huang, J., Ye, X., Yin, Y., et al. (2011). Germline competency of parthenogenetic embryonic stem cells from immature oocytes of adult mouse ovary. Hum. Mol. Genet. *20*, 1339–1352.

Melton, C., Judson, R.L., and Blelloch, R. (2010). Opposing microRNA families regulate self-renewal in mouse embryonic stem cells. Nature *463*, 621–626.

Miyoshi, N., Ishii, H., Nagano, H., Haraguchi, N., Dewi, D.L., Kano, Y., Nishikawa, S., Tanemura, M., Mimori, K., Tanaka, F., et al. (2011). Reprogramming of mouse and human cells to pluripotency using mature microRNAs. Cell Stem Cell *8*, 633–638.

Nichols, J., and Smith, A. (2009). Naive and primed pluripotent states. Cell Stem Cell *4*, 487–492.

Park, C.Y., Jeker, L.T., Carver-Moore, K., Oh, A., Liu, H.J., Cameron, R., Richards, H., Li, Z., Adler, D., Yoshinaga, Y., et al. (2012). A resource for the conditional ablation of microRNAs in the mouse. Cell Rep. *1*, 385–391.

Smith, Z.D., Nachman, I., Regev, A., and Meissner, A. (2010). Dynamic single-cell imaging of direct reprogramming reveals an early specifying event. Nat. Biotechnol. *28*, 521–526.

Somers, A., Jean, J.C., Sommer, C.A., Omari, A., Ford, C.C., Mills, J.A., Ying, L., Sommer, A.G., Jean, J.M., Smith, B.W., et al. (2010). Generation of transgene-free lung disease-specific human induced pluripotent stem cells using a single excisable lentiviral stem cell cassette. Stem Cells *28*, 1728–1740.

Srinivas, S., Watanabe, T., Lin, C.S., William, C.M., Tanabe, Y., Jessell, T.M., and Costantini, F. (2001). Cre reporter strains produced by targeted insertion of EYFP and ECFP into the ROSA26 locus. BMC Dev. Biol. *1*, 4.

Subramanyam, D., Lamouille, S., Judson, R.L., Liu, J.Y., Bucay, N., Derynck, R., and Blelloch, R. (2011). Multiple targets of miR-302 and miR-372 promote reprogramming of human fibroblasts to induced pluripotent stem cells. Nat. Biotechnol. *29*, 443–448.

Suh, N., Baehner, L., Moltzahn, F., Melton, C., Shenoy, A., Chen, J., and Blelloch, R. (2010). MicroRNA function is globally suppressed in mouse oocytes and early embryos. Curr. Biol. *20*, 271–277.

Sun, K., and Lai, E.C. (2013). Adult-specific functions of animal microRNAs. Nat. Rev. Genet. *14*, 535–548.

Takahashi, K., and Yamanaka, S. (2006). Induction of pluripotent stem cells from mouse embryonic and adult fibroblast cultures by defined factors. Cell *126*, 663–676.

Unternaehrer, J.J., Zhao, R., Kim, K., Cesana, M., Powers, J.T., Ratanasirintrawoot, S., Onder, T., Shibue, T., Weinberg, R.A., and Daley, G.Q. (2014). The epithelial-mesenchymal transition factor SNAIL paradoxically enhances reprogramming. Stem Cell Reports *3*, 691–698.

Wang, Y., Medvid, R., Melton, C., Jaenisch, R., and Blelloch, R. (2007). DGCR8 is essential for microRNA biogenesis and silencing of embryonic stem cell self-renewal. Nat. Genet. *39*, 380–385.

Wang, Y., Baskerville, S., Shenoy, A., Babiarz, J.E., Baehner, L., and Blelloch, R. (2008). Embryonic stem cell-specific microRNAs regulate the G1-S transition and promote rapid proliferation. Nat. Genet. *40*, 1478–1483.

Ying, Q.L., Stavridis, M., Griffiths, D., Li, M., and Smith, A. (2003). Conversion of embryonic stem cells into neuroectodermal precursors in adherent monoculture. Nat. Biotechnol. *21*, 183–186.

Yu, J., Vodyanik, M.A., Smuga-Otto, K., Antosiewicz-Bourget, J., Frane, J.L., Tian, S., Nie, J., Jonsdottir, G.A., Ruotti, V., Stewart, R., et al. (2007). Induced pluripotent stem cell lines derived from human somatic cells. Science *318*, 1917–1920.

Zhang, Z., Xiang, D., Heriyanto, F., Gao, Y., Qian, Z., and Wu, W.S. (2013). Dissecting the roles of miR-302/367 cluster in cellular reprogramming using TALE-based repressor and TALEN. Stem Cell Reports *1*, 218–225.

Zhao, R., Deibler, R.W., Lerou, P.H., Ballabeni, A., Heffner, G.C., Cahan, P., Unternaehrer, J.J., Kirschner, M.W., and Daley, G.Q. (2014). A nontranscriptional role for Oct4 in the regulation of mitotic entry. Proc. Natl. Acad. Sci. USA *111*, 15768–15773.

Stem Cell Reports, Volume *5*

Supplemental Information

Canonical MicroRNA Activity Facilitates but May Be Dispensable for Transcription Factor-Mediated Reprogramming

Zhong Liu, Maria Skamagki, Kitai Kim, and Rui Zhao

EXTENDED FIGURE LEGEND

Figure S1. Schematic of the canonical miRNA, endo-shRNA, mirtron, and endo-siRNA biogenesis pathways, related to Figure 1-5.

Figure S2. PCR genotyping of *Dgcr8*$^{\Delta/\Delta}$ NSC-derived iPSC clones isolated from three independent reprogramming experiments, related to Figure 2. Note that *Dgcr8*^{flox} alleles were completely disrupted in all analyzed iPSC clones. Arrow, *Dgcr8*^{flox}; arrow head, *Dgcr8*$^{\Delta}$.

Figure S3. Characterization of *Dgcr8*$^{\Delta/\Delta}$ iPSCs, related to Figure 3.

(A) The NSC-derived *Dgcr8*$^{\Delta/\Delta}$ iPSCs expressed pluripotency-associated markers (a) alkaline phosphatase, (b) SSEA-1 (red), and (c) NANOG; and (c') DAPI. Scale bars, 100 µm.

(B-C) Additional karyotypes of 40, XY *Dgcr8*$^{\Delta/\Delta}$ iPSCs.

(D) Characterization of transgene-free *Dgcr8*$^{\Delta/\Delta}$ iPSCs. (a) Bright field and (a') YFP. (b-d') The transgene-free *Dgcr8*$^{\Delta/\Delta}$ iPSCs expressed pluripotency-associated markers (b) alkaline phosphatase, (c) SSEA-1, and (d) NANOG; and (d') DAPI. Scale bars, 100 µm (white) and 50 µm (green).

Figure S1

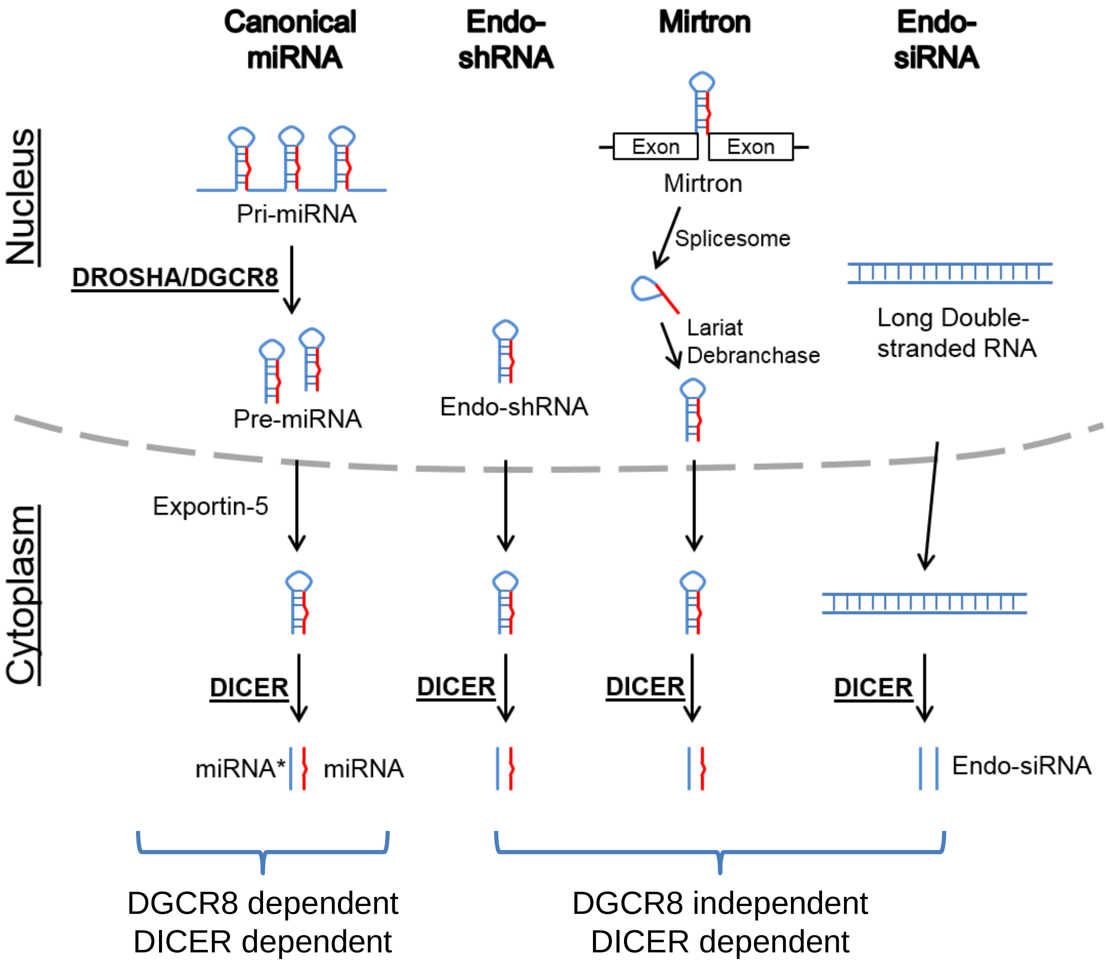

Canonical miRNA
Endo-shRNA
Mirtron
Endo-siRNA
Nucleus
Cytoplasm
Pri-miRNA
DROSHA/DGCR8
Pre-miRNA
Exon
Exon
Mirtron
Splicesome
Lariat Debranchase
Endo-shRNA
Long Double-stranded RNA
Exportin-5
DICER
DICER
DICER
DICER
miRNA*
miRNA
Endo-siRNA
DGCR8 dependent
DICER dependent
DGCR8 independent
DICER dependent

Figure S2.

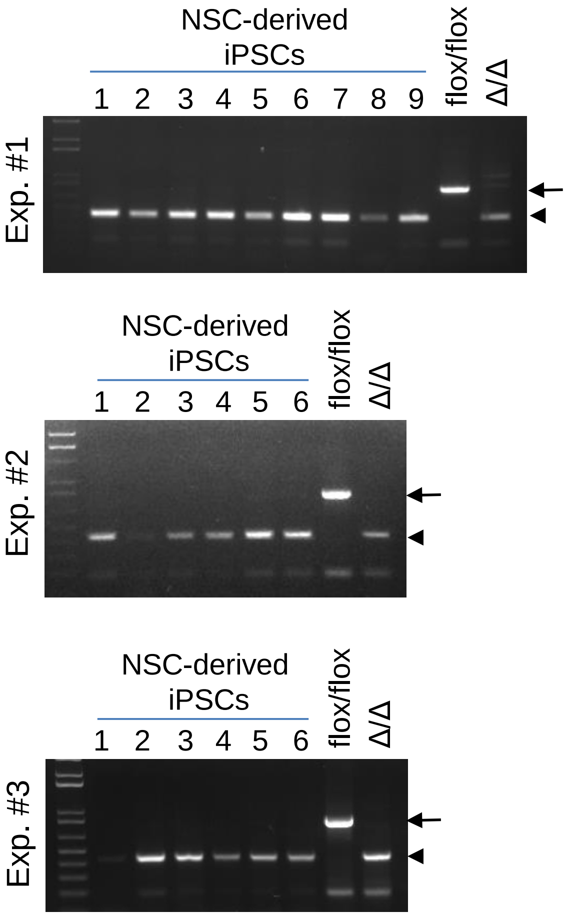
NSC-derived iPSCs
1 2 3 4 5 6 7 8 9
flox/flox
Δ/Δ
Exp. #1
NSC-derived iPSCs
1 2 3 4 5 6
flox/flox
Δ/Δ
Exp. #2
NSC-derived iPSCs
1 2 3 4 5 6
flox/flox
Δ/Δ
Exp. #3

Figure S3.

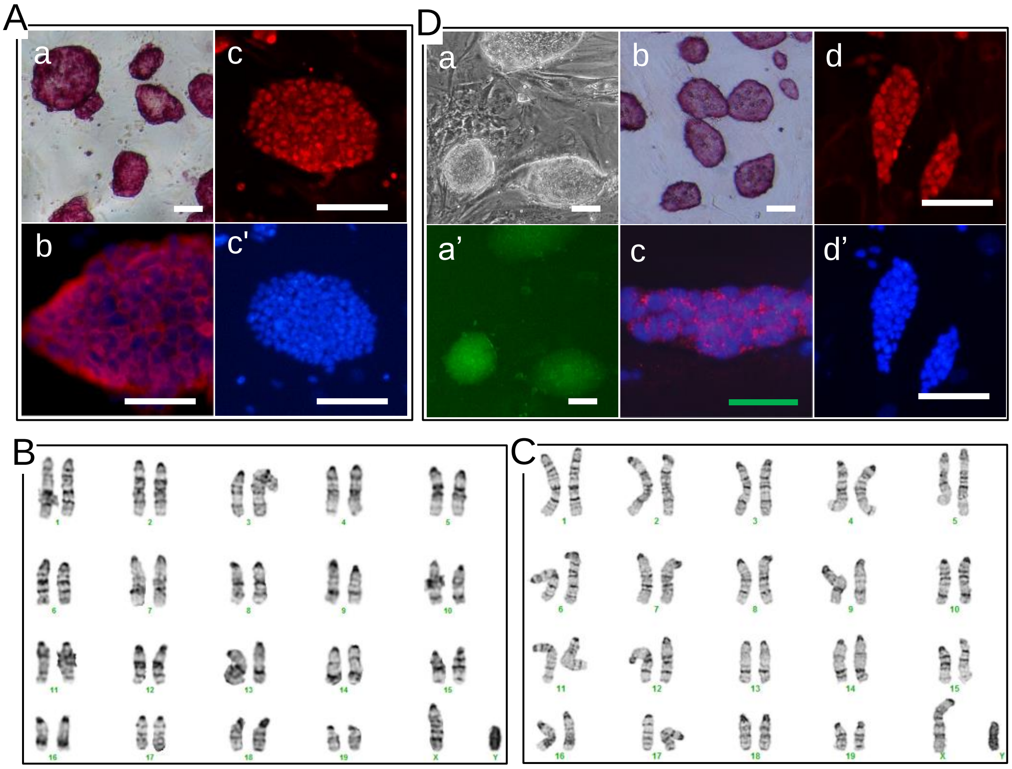

Table S1. Frequency of incomplete *Dgcr8* deletion in *Dgcr8*$^{\Delta/\Delta}$ fibroblast reprogramming, related to Figure 1.

	# of input fibroblasts	# of iPS colonies	# of *Dgcr8*$^{\Delta/\Delta}$ iPS colonies	# of incomplete deleted colonies	Reprog. efficiency (%)*
Exp. 1	50,000	2	2	0	0.004
Exp. 2	50,000	2	2	0	0.004
Exp. 3	50,000	11	9	2	0.018
Exp. 4	50,000	6	5	1	0.010
Exp. 5	50,000	1	1	0	0.002
Total		22	19	3	
% total			86.4	13.6	

*Reprogramming efficiency is determined by the ratio of *Dgcr8*$^{\Delta/\Delta}$ iPS colonies to input fibroblasts.

Table S2. Primer sequences, related to Figure 3 and 4.

Gene	Primers	Sequence
Oct4	Oct4f<br>Oct4r	5′ – AGC TGC TGA AGC AGA AGA GGA TCA – 3′<br>5′ – TCT CAT TGT TGT CGG CTT CCT CCA – 3′
Sox2	Sox2f<br>Sox2r	5′ – CAC ATG AAG GAG CAC CCG GAT TAT – 3′<br>5′ – TCC GGG AAG CGT GTA CTT ATC CTT – 3′
Nanog	Nanogf<br>Nanogr	5′ – AAC CAA AGG ATG AAG TGC AAG CGG – 3′<br>5′ – TCC AAG TTG GGT TGG TCC AAG TCT – 3′
Sox1	Sox1f<br>Sox1r	5′ – CCA AGA TGC ACA ACT CGG AGA TCA – 3′<br>5′ – ACT TGT CCT TCT TGA GCA GCG TCT – 3′
Fgf5	Fgf5f<br>Fgf5r	5′ – ATA GCA GTT TCC AGT GGA GCC CTT – 3′<br>5′ – ACT TAA CAC ACT GGC TTC GTG GGA – 3′
Krt18	Krt18f<br>Krt18r	5′ – ATC TGG AGT CAG AGC TGG CAC AAA – 3′<br>5′ – TCC AGG GCA TCG TTG AGA CTG AAA – 3′
Brachyury	Brach-f<br>Brach-r	5′ – AGC TCT CCA ACC TAT GCG GAC AAT – 3′<br>5′ – TGG TAC CAT TGC TCA CAG ACC AGA – 3′
Afp	Afp-f<br>Afp-r	5′ – AGT TTC CAG AAC CTG CCG AGA GTT – 3′<br>5′ – TGG AAG CAC TCC TCC TTG TTG TCA – 3′
Hnf4a	Hnf4a-f<br>Hnf4a-r	5′ – TTC GGC ATG GCC AAG ATT GAC AAC – 3′<br>5′ – TTG GTG CCC ATG TGT TCT TGC ATC – 3′
Eomes	Eomes-f<br>Eomes-r	5′ – AAG GCT TCC GGG ACA ACT ACG ATT – 3′<br>5′ – TGA GGC AAA GTG TTG ACA AAG GGC – 3′
Dgcr8	Dgcr8f<br>Dgcr8r	5′ – GTG GAT GAA GAG GCC TTG AA – 3′<br>5′ – TCT TGG GAG CAC AGA GAC CT – 3′
Oct4-tg	hOct4-tg-F<br>F2A-R	5′ – GCT CTC CCA TGC ATT CAA AC – 3′<br>5′ – TTG GAC CTG GAT TTG ACT CTA C – 3′
Sox2-tg	hSox2F<br>hSox2R	5′ – CAC ATG AAG GAG CAC CCG GAT TAT – 3′<br>5′ – GTT CAT GTG CGC GTA ACT GTC CAT – 3′
b-actin	bActin-f<br>bActin-r	5′ – TTG CTG ACA GGA TGC AGA AGG AGA – 3′<br>5′ – ACT CCT GCT TGC TGA TCC ACA TCT – 3′
Lentiviral transgene	hOct4-tg-F<br>hKlf4-tg-R	5′ – TGT ACT CCT CGG TCC CTT T – 3′<br>5′ – TGC TTG ACG CAG TGT CTT – 3′
Genomic control	gDgcr8-P1f<br>gDgcr8-P1r	5′ – TTT CCA ACC CAA GTC AGC AGA T – 3′<br>5′ – AGT GCA TGT GCC ATG CTG CCA – 3′
